# Research Progresses in Microstructure Designs of Flexible Pressure Sensors

**DOI:** 10.3390/polym14173670

**Published:** 2022-09-04

**Authors:** Hao Huang, Jinyao Zhong, Yongliang Ye, Renxu Wu, Bin Luo, Honglong Ning, Tian Qiu, Dongxiang Luo, Rihui Yao, Junbiao Peng

**Affiliations:** 1 State Key Laboratory of Luminescent Materials and Devices, Institute of Polymer Optoelectronic Materials and Devices, South China University of Technology, Guangzhou 510640, China; 2 Department of Intelligent Manufacturing, Wuyi University, Jiangmen 529020, China; 3 Guangzhou Key Laboratory for Clean Energy and Materials, Huangpu Hydrogen Innovation Center, School of Chemistry and Chemical Engineering, Institute of Clean Energy and Materials, Guangzhou University, Guangzhou 510006, China

**Keywords:** flexible pressure sensors, microstructures, wearable devices, electronic skin, tactile

## Abstract

Flexible electronic technology is one of the research hotspots, and numerous wearable devices have been widely used in our daily life. As an important part of wearable devices, flexible sensors can effectively detect various stimuli related to specific environments or biological species, having a very bright development prospect. Therefore, there has been lots of studies devoted to developing high-performance flexible pressure sensors. In addition to developing a variety of materials with excellent performances, the microstructure designs of materials can also effectively improve the performances of sensors, which has brought new ideas to scientists and attracted their attention increasingly. This paper will summarize the flexible pressure sensors based on material microstructure designs in recent years. The paper will mainly discuss the processing methods and characteristics of various sensors with different microstructures, and compare the advantages, disadvantages, and application scenarios of them. At the same time, the main application fields of flexible pressure sensors based on microstructure designs will be listed, and their future development and challenges will be discussed.

## 1. Introduction

Flexible electronic/wearable technology is one of the research hotspots [1,2,3,4,5,6]. According to the analysis in the report of IDTechEx, a well-known research company in the UK, the total market size of wearable products in the world is close to US $80 billion now, which has tripled in 2014, and it is predicted to reach US $138 billion in 2025 [7]. In addition, the development of electronic skin is driven by the growing interest in artificial intelligence [8], man-machine interface [9], and prosthetic skin [10]. What is more, wearable devices have great potential in health monitoring and nursing applications [11,12,13].

**Figure 1:**
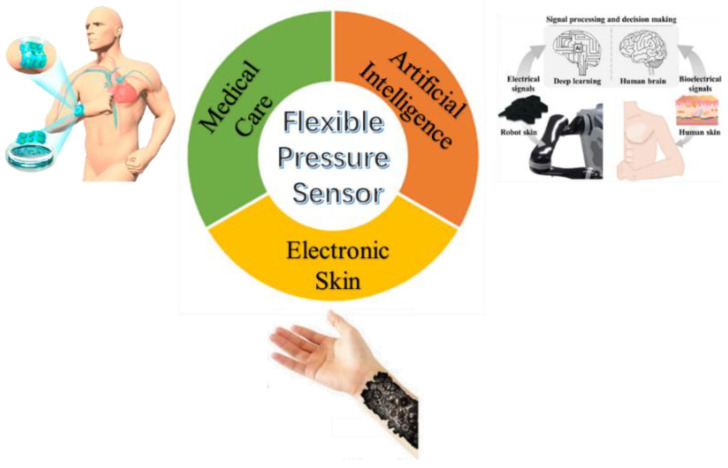
Wide application fields of flexible pressure sensors [8,16,20].

Flexible sensor is an important part of wearable devices and electronic skin [14,15]. It can effectively detect various stimuli related to specific environment or biological species, having a very bright development prospect. Among them, the research on flexible pressure sensor is the most popular, because pressure is one of the most common forms of interaction between humans and the world. Therefore, various wearable devices based on flexible pressure sensors are developing rapidly and have a wide range of applications in medical care [16,17], artificial intelligence [18], electronic skin [19,20,21], and other fields, as shown in Figure 1.

**Figure 2:**
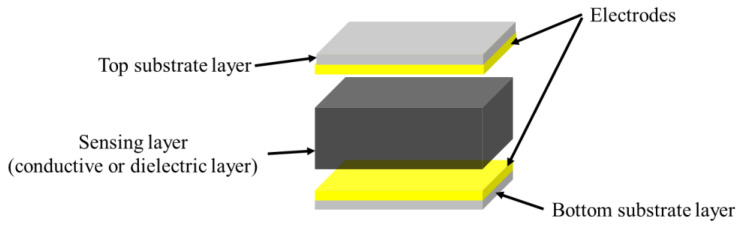
Structural diagram of flexible pressure sensor.

As shown in Figure 2, the structure of flexible pressure sensor is mainly composed of a top and bottom substrate layer, middle sensing layer (piezoresistive, capacitive, piezoelectric and triboelectric), and electrodes. After long-term research by scientists, a lot of excellent materials have been used to assemble flexible pressure sensors, such as high elastic polymer materials such as polydimethylsiloxane (PDMS) [22,23,24], polyimide (PI) [25], Ecoflex [26], and polyethylene terephthalate (PET) [27] as flexible substrate/dielectric layer, and materials with good conductivity such as graphene [28], carbon nanotubes (CNT) [29,30], MXenes [31,32], and silver nanowires (AgNWs) [33] as sensing materials and electrodes, so as to give the sensor excellent sensitivity and wide sensing range. However, with the development of society, people put forward higher requirements for the performance of flexible pressure sensor. 

So, scientists began to look for other ways to improve the performance of sensors besides looking for better materials. A large number of new studies have shown that microstructuralization of materials can also effectively improve the performances of flexible pressure sensors [34,35]. Microstructure design refers to using various processing methods to introduce small–scale microstructures such as pyramids and micro domes on the surface or inside of materials. The microstructure design of the materials can significantly improve the sensitivity, sensing range, and other characteristics of flexible pressure sensors. For example, the pyramid structure with small shape factor can significantly increase the contact area of the sensor under low pressure due to the stress concentration effect, so as to obtain a larger rate of change of electrical quantity and improve the sensitivity. Additionally, with the hierarchical fold structure, the sensor has a wide sensing range due to the continuous generation of new conductive contacts in a wide pressure range.

Therefore, the microstructure design is an important way to improve the performance of flexible pressure sensors. If we can design the material of sensor with appropriate microstructure according to different applications, we can maximize the performance of the sensor. This review classifies and analyzes the research progress of flexible pressure sensors based on microstructure design in recent years and shows the advantages and importance of material microstructure design in the field of flexible pressure sensors. Firstly, the types of flexible pressure sensors and the mechanism of microstructure are introduced, and we will demonstrate the feasibility of the introduction of microstructures to improve the sensitivity and sensing range of flexible pressure sensors. Then, we will introduce the material microstructure design of flexible pressure sensor in detail according to the structural shapes of microstructures, discuss the processing methods and characteristics of various microstructures, and compare the advantages, disadvantages, and application scenarios of different microstructures. Finally, this paper will list the main application fields of flexible pressure sensors based on microstructure design and discuss its future developments and challenges.

## 2. Sensor Types and Microstructure Mechanism

### 2.1. The Types of Flexible Pressure Sensor

According to different working principles, flexible pressure sensors can be divided into piezoresistive type, capacitive type, piezoelectric type, and triboelectric type. Their sensing principles and characteristics are shown in Table 1 [36].

### 2.2. The Mechanism of Microstructure

**Figure 3:**
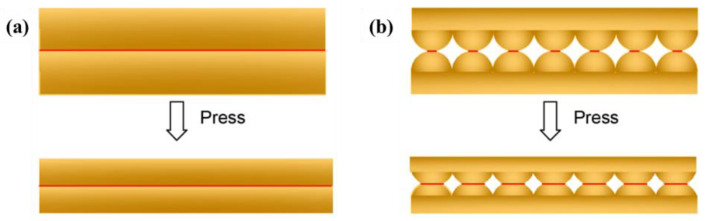
Comparison of deformation between (**a**) non micro–structured sensor and (**b**) micro-structured sensor under pressure.

When under pressure, the deformable part of the sensor will deform. For non–micro-structured sensors (Figure 3a), the pressure response comes from the compression of the conductive/dielectric layer rather than the deformation of the substrate microstructure. With the increase of pressure, the compression of the conductive/dielectric layer can only lead to a small increase in the contact area [37,38], which leads to a small change in resistance, resulting in low sensitivity of the device. For the micro-structured sensor (Figure 3b), the common essence of different microstructures that can improve the performances of the sensor is to try to reduce the Young’s modulus in mechanical properties of the device, that is, to produce the maximum deformation with the minimum force. 

**Figure 4:**
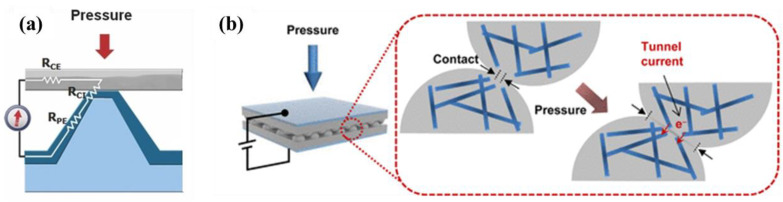
(**a**) Circuit model used to derive the sensing principle of the sensor, which relies on the change of the microstructure in response to pressure [39]. (**b**) The external pressure concentrates stress at the contact spots, which in turn causes an increase in the contact area and the tunneling currents [41].

The introduction of microstructure can cause greater deformation of the internal contact area of the sensor, resulting in more obvious changes in electrical quantities. Its internal mechanism is that the contact area will become larger, as shown in Figure 4, resulting in effects such as the increase of conductive path (Figure 4a) [39,40] and the accumulation of tunnel effect (Figure 4b) [41], so as to improve the sensitivity, sensing range, and other characteristics of the sensor.

**Figure 5:**
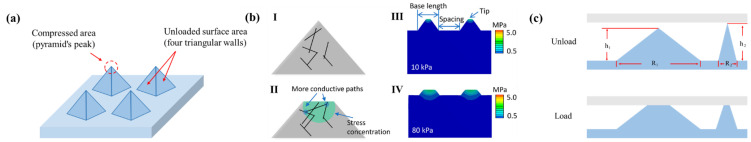
Schematic of (**a**): the definition of shape factor. (**b**): the conductive micro pyramids (**I**) without pressure and (**II**) with pressure, (**III**) under low pressure (10 kPa) and (**IV**)under high pressure (80 kPa) to show that the stress concentrated at the pyramid tips [43]. (**c**): comparison of microstructure with different shape factors (aspect ratio) before and after load.

At the same time, researchers have defined the shape factor of microstructure, that is, the ratio of compressed area (e.g., the pyramid’s peak) to the total unloaded surface area of free expansion (e.g., the four triangular walls), as shown in Figure 5a [39]. Under the same pressure, the smaller the shape factor is, the easier the microstructure is to deform. Then, the contact resistance or capacitance changes significantly under low pressure, and the sensor has high sensitivity [39,42]. As shown in Figure 5b,c, this is because the microstructure with small shape factor (Figure 5c right) actually has a high aspect ratio (h/R value). Affected by the stress concentration effect, the pressure applied on the sensor is concentrated at the tip of the microstructure [43]. Through computer simulation, with the increase of the pressure, the height of the microstructure with small shape factor (high aspect ratio) will sharply decrease and the contact area will greatly increase, as shown in the figure. Therefore, the conductive path can significantly increase under micro pressure, thereby improving the sensitivity of the sensor [44]. Correspondingly, the microstructure with large shape factor (Figure 5c left) has low aspect ratio, poor stress concentration effect, and low sensitivity under low pressure, but it can continuously increase the contact area and conductive path in a wide pressure range, resulting in the improvement of the sensing range.

## 3. Microstructure Design

Because the microstructure of materials can effectively improve the performance of sensors, there has been lots of studies devoted to the microstructure design of flexible pressure sensors. 

On the one hand, the inspiration of the microstructure comes from artificial design. Researchers study, simulate, and optimize the mechanical structures on the macro level with large Young’s modulus which is easy to deform under pressure, and then migrate them to the micro scale of the sensor to realize the microstructure design of the sensor. This kind of microstructure is generally uniform and regular, such as pyramids, micro columns, micro domes, and other structures.

On the other hand, the inspiration comes from bionic design. After billions of years of evolution, organisms often form fine and precise biological structures in order to survive in various harsh environmental conditions, such as the hillock structure of lotus leaves, the slit at the spider’s leg joints, and the interlocking spinous process structure on human skin. So, rich and complex microstructures can also be obtained by using natural materials as template directly or imitating them indirectly.

We divide the abundant microstructures obtained by different methods into six categories according to their structural shapes, including sharp bulge microstructures, micro fluctuation structures, wave/ridge microstructures, hierarchical microstructures, composite microstructures, and porous microstructures. Different microstructures have different shape features, which endow flexible pressure sensors with different sensitivity, sensing range, detection limit, and other characteristics.

### 3.1. Sharp Bulge Microstructures 

According to the mechanism of microstructures, researchers will first choose microstructures with high aspect ratio, such as pyramids, hills, cilia, and other microstructures, in order to obtain flexible sensors with high sensitivity. Due to their high aspect ratio, these microstructures have small shape factors, which can significantly improve the sensitivity of the sensor.

**Figure 6:**
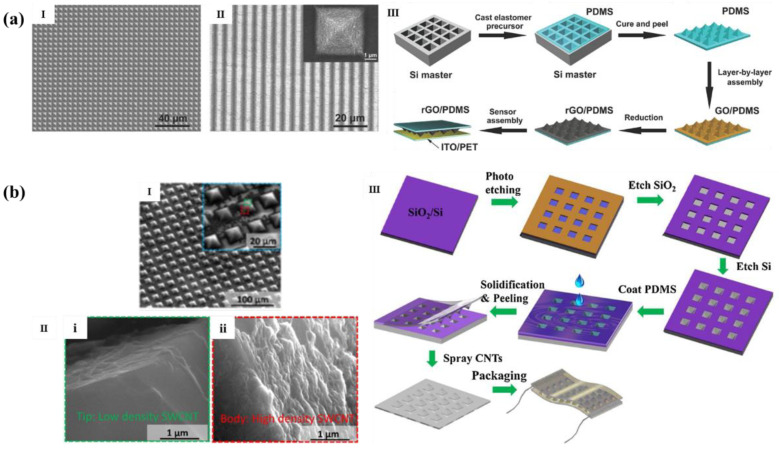
(**a**,**I**) SEM image of PDMS with uniform pyramid pattern array; (**II**) inclined SEM image of the microstructural PDMS film covered with graphene; (**III**) schematic diagram of tactile sensor manufacturing process [45]. (**b**,**I**) 45° SEM view of PDMS with uniform pyramid array; (**II**) SEM image of pyramid with (**i**) low-density SWCNT layer and (**ii**) high-density SWCNT layer; (**III**) manufacturing process of flexible pressure sensor [46].

Pyramid microstructures are generally obtained by photolithography template method. Zhu et al. [45] coated PDMS on the photolithographic silicon template to obtain a uniform pyramid microstructure substrate with a size of less than 4.5 µm after depositing a layer of reduced graphene oxide (RGO), as shown in Figure 6a, which was part of a piezoresistive flexible pressure sensor with 5.53 kPa^−1^ high sensitivity under 100 Pa. 

This kind of sensor has smaller microstructure size and more obvious deformation than unstructured one under the same pressure, which also has low pressure strong detection lower limit (2 Pa) and low response time (0.2 ms). And as shown in Figure 6b, Huang et al. [46] sprayed single wall carbon nanotubes (SWCNT) on the pyramid microstructure PDMS film obtained from the photolithographic silicon template repeatedly, which greatly improved the conductivity of the sensor and obtained a sensor with extremely high sensitivity (1907.2 kPa^−1^, <400 Pa; 8655.6 kPa^−1^, 400–900 Pa; 1874.5 kPa^−1^, >900 Pa).

At the same time, scientists also found hillock–like sharp bulge microstructures with small shape factor from natural materials (such as lotus leaves, roses, etc.). So, researchers exploited natural materials as templates to prepare the substrate of hillock–like microstructure, so as to obtain a flexible pressure sensor with high sensitivity at a low cost.

**Figure 7:**
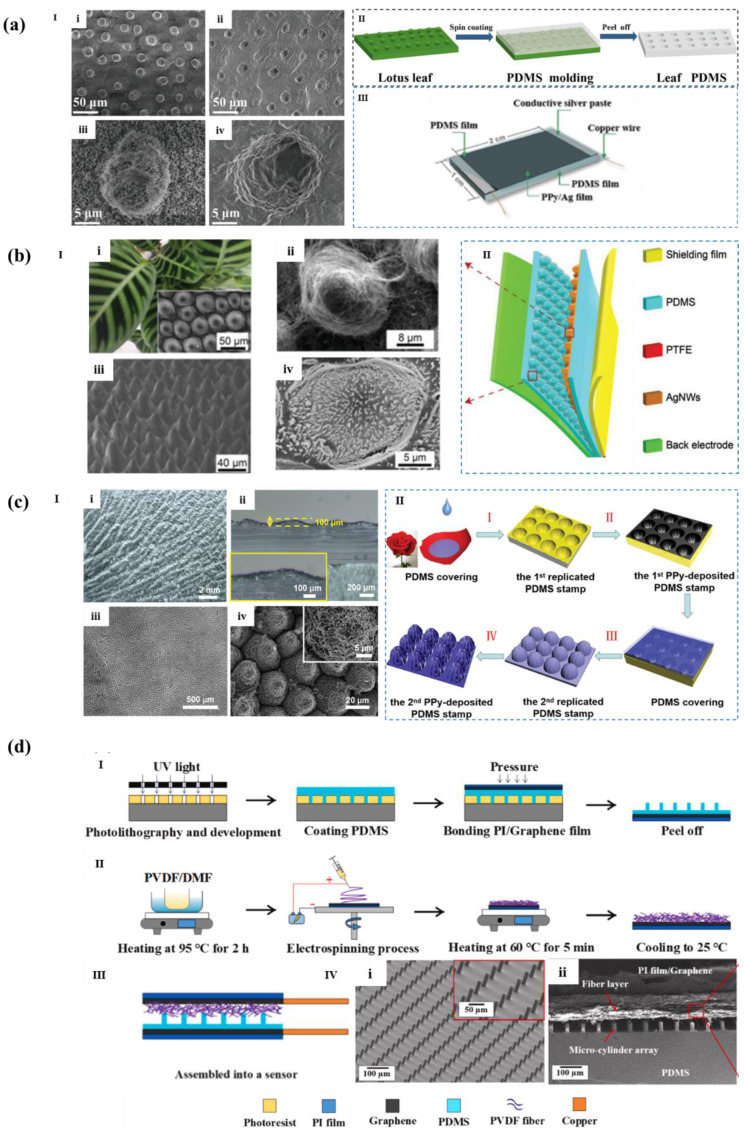
(**a**,**I**) SEM images: (**i**,**ii**) lotus leaf; (**iii**,**iv**) microstructural PDMS films; (**II**) The manufacturing process of lotus leaf-like sharp bulge patterned PDMS film; (**III**) The structure of flexible pressure sensor [47]. (**b**,**I**) SEM images: (**i**) C. Zebrine leaves; (**ii**,**iii**,**iv**) bionic microstructures. **II**) The structure of Teng e-skin flexible pressure sensor [48]. (**c**,**I**) image of rose-like sharp bulge microstructure: (**i**,**ii**) optical images; (**iii**,**iv**) SEM images. (**II**) Manufacturing process of rose-like sharp bulge microstructural PDMS film [49]. (**d**) Schematics of (**I**,**II**) the fabrication process of microstructured electrodes; (**III**) the assembled array as a capacitive pressure sensor; (**IV**) SEM images of the composite dielectric layer. (**i**) 45° tilt-view, (**ii**) Cross-sectional view [52].

Wang et al. [47] re-engraved the lotus leaf surface microstructure on PDMS, and then manufactured a sensitive sensor integrating strain and pressure sensing by sandwiching polypyrrole (PPy)/Ag hybrid film between two micro pattern PDMS substrates, as shown in Figure 7a. The sensitivity of the flexible sensor can reach about 0.58 kPa^−1^ in the range of 200–300 Pa. Yao et al. [48] applied the Calathea Zebrine blade to the manufacture of triboelectric sensor and used the microstructure of the blade as a template to construct a friction layer with interlocking structure, as shown in Figure 7b. The results show that the sensitivity (127.22 mV kPa^−1^) of the flexible pressure sensor with bionic microstructure is 14 times higher than that of the sensor with flat friction layer, with high cycle stability (5000 cycles). In addition, as shown in Figure 7c, Yu et al. [49] re-etched PDMS substrate with rose microstructure by secondary template method, and deposited PPy active layer on the surface to form the sensor. The hillock structure on the surface of PDMS combined with the wrinkle pattern of PPy film gives the sensor ultra-high sensitivity (70 kPa^−1^, <0.5 kPa), ultra-low detection limit (0.88 Pa), wide pressure detection range (32 kPa), and rapid response time (30 ms). Moreover, due to the photoelectric characteristics of PPy, the sensitivity of the sensor is increased to 120 kPa^−1^ (<0.5 kPa), and the lower detection limit is 0.41 Pa under illumination.

In addition, scientists also found that the ciliary microstructure in animals has a high aspect ratio, that is, a small shape factor, which can be used as a sensory organelle to continuously monitor the environment and play an important role in chemical sensing, environmental adaptation, and signal transduction, [50] and especially can effectively sense the small vibration in the surrounding environment [51]. Therefore, there has been numerous research studies that use this bionic idea to introduce the ciliary microstructure into the flexible sensor to reduce the minimum detection limit of the sensor. Lin et al. [52] developed a dual-layer dielectric structure comprising a layer of electro spun fiber and an cilia-like array of microcylinders prepared by photolithography and used it to fabricate a novel high-sensitivity capacitive pressure sensor, as shown in Figure 7d. It had high sensitivity of 0.6 kPa^−1^, rapid response time of 25 ms, and ultralow limit of detection of 0.065 Pa.

Table 2 lists the performances of sensors with sharp bulge microstructures. Flexible pressure sensors with sharp bulge microstructures always have ultra-high sensitivity under low pressure because they have obvious deformation under low pressure. This kind of sensor is suitable for monitoring weak signals, such as breathing, pulse, etc.

### 3.2. Micro Fluctuation Structures

Micro fluctuation is also a common microstructure. It fluctuates more slowly than sharp bulge microstructure, but it also has small shape factor like sharp bulge microstructure. Therefore, the micro fluctuation structure is also expected to improve the sensitivity of pressure sensors. 

**Figure 8:**
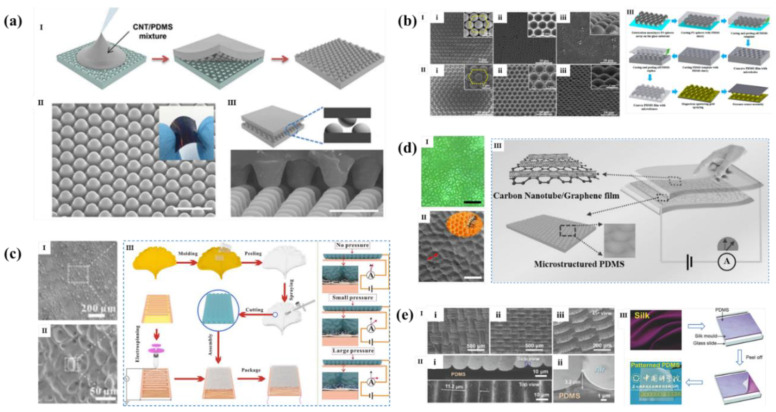
(**a**) Micro structured sensor with interlocking micro dome array. (**I)** manufacturing process of micro dome array; (**II**) Inclined SEM image of micro dome structure; (**III**) Schematic diagram of interlocking structure (upper) and cross-sectional SEM image (lower) [41]. (**b**) Flexible pressure sensor based on micro dome. (**I**) SEM images of the monolayer PS sphere arrays fabricated by 2 μm PS microspheres (**i**), the concave PDMS film (**ii**), and the PDMS surface with micro dome patterns (**iii**). The insets show enlarged images of the corresponding orthogonal structures; (**II**) Change the above size to 5.6 μm; (**III**) Manufacturing process of micro dome array and flexible pressure sensor [64] (**c**). (**I**,**II**) SEM image of bionic ginkgo biloba leaf–like micro fluctuation structure; (**III**) schematic diagram of manufacturing process and sensing mechanism of bionic piezoresistive sensor based on MXene [65]. (**d**,**I**) Optical image of an epipremnum aureum leaf surface; (**II**) SEM image of m-PDMS films; (**III**) The structure of ACNT/G pressure sensor [66]. (**e**,**I**) SEM images of patterned (**i**) L-PDMS and (**ii**) H-PDMS films and (**iii**) 45° views of H-PDMS; (**II**,**i**) Top and side views of H-PDMS, (**ii**) High magnification SEM images of (**i**); (**III**) Schematic diagram of manufacturing process of flexible patterned PDMS film [67].

Micro dome is the most common micro fluctuation structure. Park et al. [41] coated CNT/PDMS on the concave dome silicon template made by photolithography to obtain a resistance sensor of CNT/PDMS interlocking micro domes array, as shown in Figure 8a. The interlocking micro dome can cause stress concentration at the contact point of the dome when small pressure was acted on it and produce local deformation to increase the contact area, which can reduce the tunneling resistance and improve the sensitivity (15.1 kPa^−1^, <500 Pa). What is more, as shown in Figure 8b, Zhang et al. [64] employed closely arranged PS microsphere arrays as sacrificial template to cover the PDMS on their surface. After curing, the PS microsphere was removed with tetrahydrofuran (THF) solution to obtain the PDMS reverse template with bowl shaped concave microstructure. With this template, the PDMS substrate with micro-dome structures was obtained, and finally a piezoresistive pressure sensor with the sensitivity of 15 kPa^−1^ under 100 Pa and 2 kPa^−1^ at a pressure of 100–400 Pa.

Scientists also found micro fluctuation structures similar to hillocks from natural materials, but they fluctuate more slowly, have more irregular distribution and different shapes than hillocks. In nature, ginkgo biloba leaves and epipremnum aureum leaves have micro fluctuation structures, so they are widely studied and applied in flexible pressure sensors, as shown in Figure 8c,d.

Yan et al. [65] re-engraved the surface of Ginkgo biloba leaves by template method to obtain a patterned PDMS film, and then sprayed Mxene on the film (Figure 8c). In order to further improve the sensitivity of the sensor, a layer of poly vinyl alcohol (PVA) fiber was inserted between the conductive layer and the interdigital electrode by electrospinning. The sensor had an ultra-high sensitivity of 403.46 kPa^−1^, a fast response time of 99.3 ms, and an ultra-low detectable pressure limit of 0.88 Pa. At the same time, the sensor has more than 12,000 loading/unloading cycles stability. Jian et al. [66] used epipremnum aureum leaf template to make microstructure PDMS (m–PDMS), and then used chemical vapor deposition (CVD) to directly grow highly conductive aligned carbon nanotubes/graphene (ACNT/G) films on m–PDMS films (Figure 8d). Finally, two ACNT/G/PDMS films were placed face-to-face to construct a flexible ACNT/G pressure sensor. Due to the unique layered structure of ACNT/G and m–PDMS films, the obtained pressure sensor showed high sensitivity (19.8 kPa^−1^, <0.3 kPa), low detection limit (0.6 Pa), fast response time (<16.7 ms), and excellent stability of more than 35,000 cycles.

In addition, researchers also use artificial templates to get micro fluctuation structures, such as silk with a rough surface. As shown in Figure 8e, Wang et al. [67] used silk as a template and coated PDMS on the silk template to obtain a micro convex structure. After attaching single wall nanotubes (SWNTs) to its surface, a piezoresistive pressure sensor can be made, which had a sensitivity of 1.80 kPa^−1^ (<300 Pa), a low detection limit of 0.6 Pa, and a stability of more than 67,500 cycles.

Table 3 lists the performances of sensors with micro fluctuation structures. Because the shape factor of the micro fluctuation structure is small, the sensor has high sensitivity under low pressure, just like the sharp bulge microstructure. However, both of them have the problems of stress dispersion and low deformation under high pressure with small shape factor, so the sensing range of the sensor is narrow. Therefore, the flexible pressure sensor with these two kinds of microstructures is suitable for the field of small pressure sensing.

### 3.3. Wave/Ridge Microstructures

In order to solve the problem of low sensitivity caused by stress dispersion and low deformation of the above microstructures under high pressure, scientists have designed a microstructure that fluctuates or bulges in only one direction on the surface of the material, which is similar to the shape of wave or ridge. The wave/ridge microstructures can concentrate stress under high pressure, improve the deformation degree of the material under high pressure, and thus increase the response range to pressure.

Wave/ridge microstructures are generally obtained by pre–stretching/ultraviolet (UV) exposure method. It refers to pre–stretching the flexible substrate and then exposing it under UV in an ozone atmosphere to form a silicon oxide thin layer on its surface. When the flexible substrate is released to its initial state of zero strain, the rigid surface does not shrink synchronously with the interior due to the modulus mismatch, resulting in wave-like or ridged microarray structures on the surfaces.

**Figure 9:**
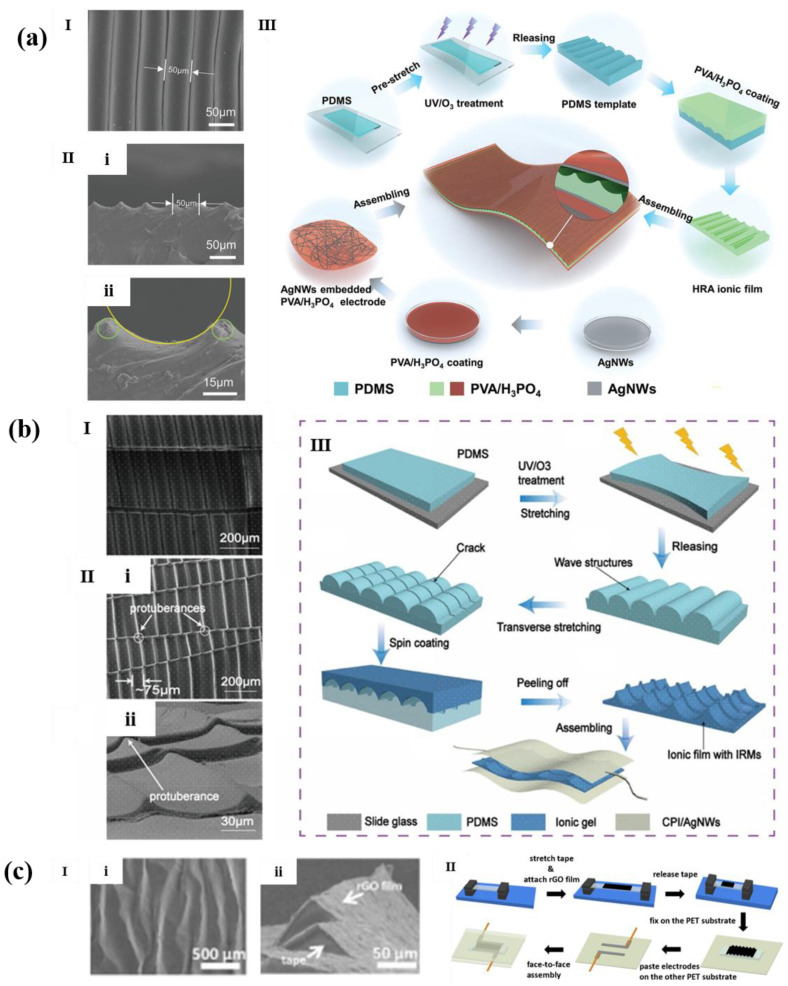
(**a**,**I**) SEM image of the patterned PDMS template; (**II**,**i**) A cross-sectional view of the templated PVA/H_3_PO_4_ HRA film, and (**ii**) contour detail of HRA; (**III**) The fabrication procedure for the pressure sensor [79]. (**b**,**I**) SEM image of the PDMS template with 40% pre-stretching; (**II**) SEM image of (**i**) the dielectric layer with IRM and (**ii**) its tilt-view; (**III**) Schematic illustration of the procedures of the sensor [80]. (**c**,**I**) SEM image of (**i**)the surface of wrinkled structure, (**ii**) the cross-section of the wrinkled structure; (**II**) Fabrication process of the rGO-based pressure sensor with wrinkled structure [81].

Qin et al. [79] used pre-stretching/UV exposure method to obtain a PDMS substrate with ridge microstructure, and then poured and solidified PVA/H_3_ PO_4_ solution on the substrate, then the hill-ridge architecture (HRA) iontronic film of PVA/H_3_ PO_4_ with sinusoidal folds formed, as shown in Figure 9a. Then, using PVA/H_3_ PO_4_/AgNWs as flexible electrode, the capacitive pressure sensor was assembled. The sensor has the advantages of high sensitivity (37.78 kPa^−1^ (<4 kPa)), fast response and recovery time (23 ms/11 ms), wide sensing range (350 kPa), and high mechanical stability. 

Additionally, as shown in Figure 9b, Zou et al. [80] used the same method to produce wave microstructures on the surface of PDMS. Then, transverse cracks were made on the wave microstructure by transverse tension, which further improved the force sensitivity of the sensor. Then, the ionic gel was spin coated to the wave microstructures as the dielectric layer, and CPI/AgNws was used as the electrode to assemble a flexible sensor with CPI/AgNWs/Ionic Dielectric Layer/AgNWs/CPI sandwich structure. The sensor has a wide sensing range (300 kPa), high sensitivity (92 kPa^−1^, <400 Pa), and a minimum detection limit of 1 Pa.

The human skin is like a mechanical sensor that combines sensitivity and linearity. For example, the skin of a human fingertip has ridges that can amplify subtle external stimuli. So, numerous studies have developed bionic wave/ridge microstructures inspired by human skin. As shown in Figure 9c, Weng et al. [81], inspired by the epidermal ridges of human fingertips, fabricated rGO/tape films with bionic wavy structure through simple pre-stretching/UV exposure method, and then fixed them on a PET substrate. Finally, the wrinkled rGO structure (wrGOs) pressure sensor was fabricated by face-to-face assembly of PET substrate with wrGOs/tape composite material and PET substrate with electrode, whose sensitivity of the sensor is as high as 5.77 kPa^−1^ (0–490 Pa). In addition, the response/recovery time is about 97 ms and 98 ms, and the pressure detection limit is as low as 3 Pa.

Table 4 lists the performances of some sensors with wave/ridge microstructures. Compared with the previous two microstructures, the wave/ridge microstructure has a larger shape factor and will reach saturation under higher pressure, so the flexible pressure sensor can have a wider sensing range.

### 3.4. Hierarchical Microstructures

Although regular geometry microstructures can improve the sensitivity of flexible pressure sensors to a certain extent, they are generally uniform and have no hierarchical change effect, resulting in high sensitivity that can only be maintained in the range of low and medium pressure. In order to further improve the sensing range of sensors, people usually process hierarchical microstructures. This kind of microstructure consists of the same shape units, but these units have different height and other parameters, so that when the pressure gradually increases, the microstructures at different levels of the sensor can be activated and deformed in turn, widening the channel of electrical characteristics change under high pressure, so as to improve the sensitivity performance under high pressure. 

**Figure 10:**
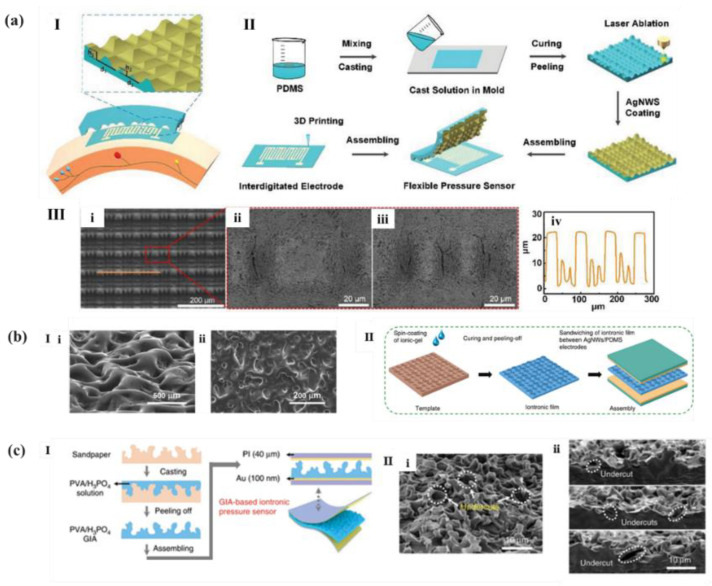
(**a**,**I**) Schematic illustration of the flexible pressure sensor; (**II**) The fabrication process of flexible pressure sensor; (**III**) SEM images of laser scribed PDMS surface [84]. (**b**,**I**) The FESEM images of the inclined view (**i**) and top view (**ii**) of the microstructured iontronic film; (**II**) A schematic of the fabrication sequence [86]. (**c**,**I**) Schematic illustration of the preparation of a GIA-based iontronic pressure sensor; (**II**) A 45° tilt-view (**i**) and cross-sectional (**ii**) view SEM images of the film [87].

Du et al. [84] obtained the hierarchical sharp bulge-like microstructures by using a femtosecond laser with a wavelength of 355 nm to ablate the flat PDMS film, as shown in Figure 10a. The first order microstructure was defined by grid line scribing while the second order microstructure was patterned by the straight-line scribing. Then, combining the silver nanowires coated, laser-ablated hierarchical microstructured PDMS, and an interdigital electrode to get the flexible sensor, with high sensitivity of 4.48 kPa^−1^ and a wide detection range from 0 to 65 kPa. 

**Figure 11:**
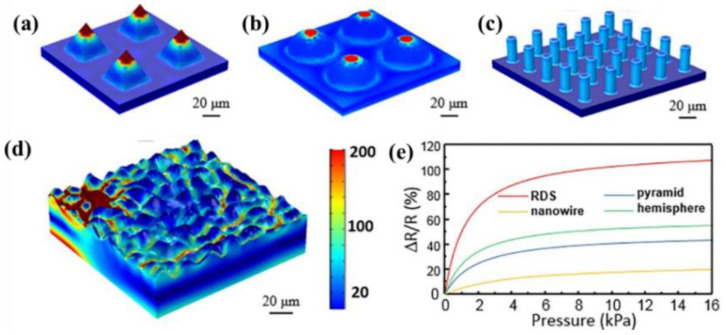
The pressure distribution of (**a**) pyramid, (**b**) hemisphere, (**c**) column, and (**d**) fold microstructures under 5 kPa load, and (**e**) their test results [85].

In addition, scientists usually use artificial material templates with coarser or hierarchical surfaces, such as sandpaper, to get more varied hierarchical fold structures. As shown in Figure 11, Pang et al. [85] analyzed the pressure distribution of pyramid (Figure 11a), hemisphere (Figure 11b), column (Figure 11c), and fold (Figure 11d) microstructures under 5 kPa load. The stress of the fold microstructure was concentrated on the initial contact peak and can be transmitted to the root of the adjacent peak. The change of the contact area was small in the range of low pressure. Although the initial contact point of the fold structure first showed saturation under a certain pressure, new contact points would be generated with the increase of pressure to compensate for the change of the whole resistance. Therefore, the sensor still had high sensitivity in a large pressure range. So, the hierarchical fold microstructure helped to broaden the sensing range of the sensor. 

As shown in Figure 10b, Chhetry et al. [86] used room temperature ionic liquid 1-ethyl−3–methylimidazolium bis(trifluoromethylsulfonyl)imide ([EMIM][TFSI]) and poly(vinylidene fluoride–co–hexafluoropropylene) (P(VDF-HFP)) polymer to prepare ion ionization films with high interface capacitance, and then grafted the randomly distributed microstructure on SiC sandpaper onto ion ionization film. Then, toke the film with irregular microstructure as the dielectric layer, the sensor had high sensitivity (131.5 kPa^−1^, <1.5 kPa; 11.73 kPa^−1^, 5–27.7 kPa) under a wide range and a detection limit of 1.12 Pa. In addition, as shown in Figure 10c, Bai et al. [87] used sandpaper as a template to make PVA/H_3_ PO_4_ with gradual filling fold microstructure as the dielectric layer. These uneven protrusions can ensure that the structure can be effectively compressed under high pressure. Finally, a capacitive flexible pressure sensor with wide linear detection range and high sensitivity (3302.9 kPa^−1^, <10 kPa; 671.7 kPa^−1^, 10–100 kPa; 229.9 kPa^−1^, 100–360 kPa) was obtained. In addition, the sensor had a detection limit of 0.08 Pa. Table 5 lists the performances of some sensors with hierarchical microstructure. The layered microstructure with more disordered distribution can realize layered deformation under gradually increasing pressure. Thus, flexible pressure sensor based on layered microstructure realizes a wide linear detection range and is suitable for the field of large pressure sensing.

### 3.5. Composite Microstructures

The performance improvement of flexible sensor is limited by a single microstructure. Sharp bulge microstructure and micro fluctuation structure mainly improve the sensitivity of the sensor, while wave/ridge microstructure mainly improve the sensing range of the sensor. In addition to the hierarchical design of a single microstructure, a large number of research studies have also tried to combine the above single microstructures to form composite microstructures, so that the sensor can also be equipped with hierarchical changes, better improving the sensitivity and sensing range of the sensor. According to Archard theory [97], compared with single microstructure, the introduction of composite structure can further enhance the linearity of pressure sensor. Researchers have used laser direct writing (LDW)/laser grid marking (LGM), photolithography, and natural material template method to obtain composite microstructures.

**Figure 12:**
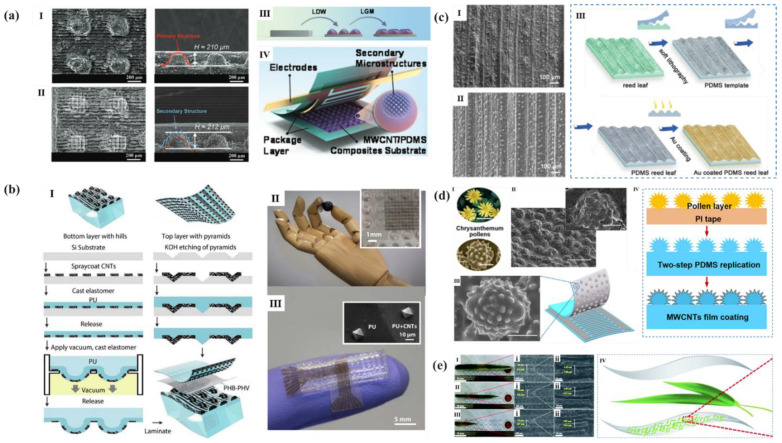
(**a**): Top and side views of SEM images of (**I**) SLMs and (**II**) HMs; (**III**) The sandwiched structure of the flexible piezoresistive sensor; (**IV**) fabrication of HMs [98]. (**b**) E-skin fabrication and appearance. (**I**) E-skin fabrication and assembly; (**I**) Optical image of a fabricated e-skin and close-up view on the hills and electrodes; (**III**) Optical image showing the CNT–PU interconnects for signal recording with LCR meter and SEM picture of the top e-skin layer with molded pyramids [99]. (**c**,**I**) SEM image of the natural reed leaf; (**II**) SEM image of the PDMS reed leaf; (**IV**) The fabrication of the micronanostructured capacitive sensor using a natural reed leaf as the template [100]. (**d**,**I**) Photograph of wild and SEM image of the hierarchical structure of the pollen grain; (**II**) Side-view SEM image of the hierarchical structure-patterned PDMS surface; (**III**) Schematic diagram of a piezoresistive sensor composed of MWCNT/PDMS with pollen-shaped hierarchical structures. (**IV**) Preparation process flow of the hierarchical structure-patterned film [101]. (**e**) Real photographs and corresponding SEM images of natural bamboo leaves at senile (**I**), mature (**II**), and infantile stages (**III**); (**IV**) Structure diagram of flexible pressure sensor [102].

Zhang et al. [98] processed MWCNT/PDMS substrate by LDW technology to obtain single-layer microstructures (SLMs) firstly, and then processed by LGM technology to obtain uniformly distributed but height-different microgrids structure on the surface of SLMs to obtain composite hierarchical microstructures (HMs), as shown in Figure 12a. The piezoresistive pressure sensor based on this substrate had a wide pressure sensing range (0.90 kPa^−1^, <600 Pa; 11.06 kPa^−1^, 600 Pa–10 kPa; 4.5 kPa^−1^, 10–300 kPa). In order to simulate the multi-level interlocking structure in human skin, Boutry et al. [99] designed a substrate microstructure combining domes and pyramids by photolithography, which was used to make capacitive electronic skin, as shown in Figure 12b. In addition, inspired by the sunflower, researchers spiraled the pyramid microstructure according to the leaf order, which improved the sensitivity and cycle stability of the sensor. The sensor could be applied to pressure and strain at the same time, whose sensitivity to normal force and tangential force could reach 0.19 ± 0.07 kPa^−1^ and 3.0 ± 0.5 kPa^−1^, respectively. The response time was within milliseconds and had excellent cycle stability (more than 30,000 cycles). 

At the same time, there are plenty of composite microstructures composed of two structures in natural materials, such as micro groove/sharp bulge composite structure, micro dome/cone composite structure, and so on, as shown in Figure 12c–e.

Natural reed leaves show layered and anisotropic microstructures. Scientists can clearly identify the periodically distributed micro grooves with an average height of 150 μm from the reed leaves, and the randomly distributed sharp bulge microstructures can be observed along the grooves. Liu et al. [100] re engraved reed leaf microstructure on PDMS by secondary template method, and plated gold layers on its front and back by physical vapor deposition (PVD) to form a capacitive sensor (Figure 12c). The sensor had high sensitivity (0.6 kPa^−1^), lower detection limit (4.5 Pa), fast response/recovery time (180/120 ms), and the sensing range was 0 to 40 kPa. The pollen grains of wild chrysanthemum have a special composite structure. There are conical secondary features on the hemispherical structure, so that they can firmly adhere to the stigma. Zhao et al. [101] prepared a master mold with a pollen by assembling pollen grains on the PI tape using a blade coating method. Then, a PDMS film based on pollen composite microstructure was obtained by secondary template method (Figure 12d). Using MWCNT as conductive layer, the micro structured MWCNT/PDMS film and interdigital electrode were placed face-to-face to assemble a pressure sensor. The sensor showed a high sensitivity of 3.5 kPa^−1^ in a wide linear response range of 0 to 218 kPa, and the linear fitting coefficient (R^2^) reaches 0.997, indicating good linearity. In addition, the sensor has good cycle stability (10,000 cycles) and fast response/recovery time (31 and 52 ms). Liu et al. [102] observed that the surface of bamboo leaves had a composite microstructure, which is composed of mound like protrusions and grooves, and its microstructure size was affected by the maturity of leaves (Figure 12e). The researchers assembled a capacitive pressure sensor with PDMS as the substrate, AgNWs/MXene as the electrode, and dry bamboo leaves as the dielectric layer. The sensor with aged bamboo leaves had the highest sensitivity. It has a high sensitivity of 2.08 kPa^−1^ (<1 kPa), a wide detection range of up to 600 kPa, and a good stability of more than 4000 cycles.

Table 6 summarizes the performances of some sensors with composite microstructures. Because the composite microstructures also have hierarchical changes, the linearity of the sensors are good, and the sensitivities are stable in a wide range. They can be applied to large pressure sensing or fields requiring high sensing stability.

### 3.6. Porous Microstructures

In addition to sensitivity and sensing range, in some scenarios, we may also need to obtain flexible pressure sensors with high compressibility and low density. To meet this need, scientists often use flexible materials with internal porous microstructures as conductive or dielectric layers to manufacture lightweight pressure sensors. In addition, because flexible porous materials are more prone to deformation under low pressure, the detection limits of pressure sensors based on them are generally low.

Scientists often obtain porous microstructures by sacrificing templates methods or using sponges, artificial foam, and other materials as templates. The sacrificial template method is usually to coat all the surface of the sacrificial template with the material used first, and then remove the template by dissolving after waiting for curing. This method can dig holes inside material without damaging the surface of the material. We can obtain porous microstructures with different shapes or sizes by controlling the shape or size of the basic units that make up the sacrificial template [107].

**Figure 13:**
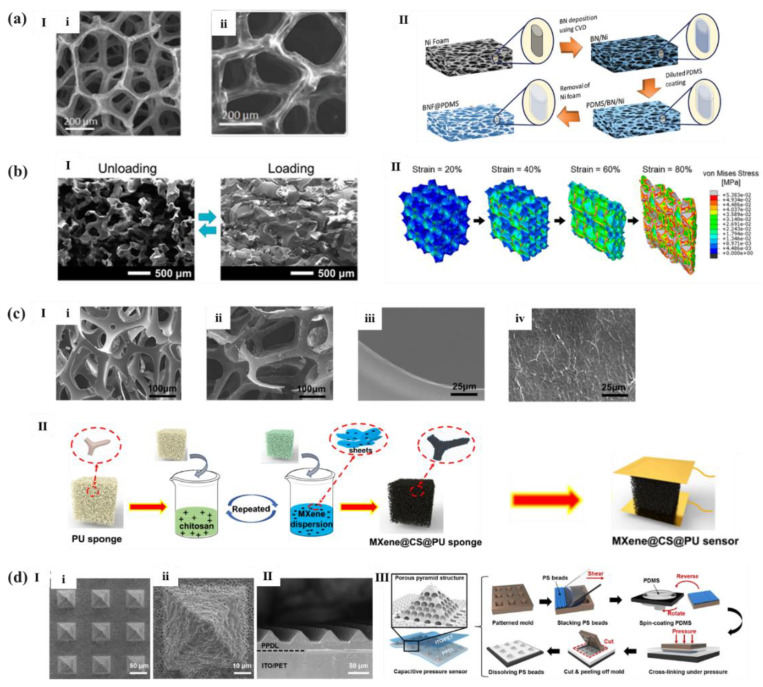
(**a**): (**I**) SEM image of a free-standing BNF (**i**) and its corresponding EDX elemental mappings of the BNF/PDMS (**ii**); (**II**) Schematic of the fabrication process of BNF/PDMS [107]. (**b**,**I**) SEM images of the porous Ecoflex dielectric layer before and after being compressed; (**II**) Gradual compressive behavior of the representative model of the porous Ecoflex dielectric cube [108]. (**c**,**I**) SEM images of the backbones of neat PU sponge (**i**,**iii**) and of MXene/CS/PU sponge (**ii**,**iv**); (**II**) Schematic illustrating the fabrication process of MXene/CS/PU sponge sensor [109]. (**d**,**I**) The top view of SEM images of the PPDL; (**II**) The cross-sectional view of SEM images of the PPDL; (**III**) Schematic depiction of PPDL fabrication process [110].

Tay et al. [107] used foam nickel as sacrificial template, where growing hexagonal boron nitride (h-BN) by CVD method, and then immersed BN/Ni foam in PDMS solution, cured and dried, as shown in Figure 13a. Finally, they removed Ni foam template with hydrochloric acid and obtained light and highly elastic hexagonal boron nitride foam (BNF)/PDMS dielectric layer, fabricating a super elastic lightweight pressure sensor with a low detection limit of less than 1 Pa and a sensitivity of 0.854 kPa^−1^ at less than 500 Pa and 0.29 kPa^−1^ at 550 Pa–2.1 kPa. What is more, sugar cubes are also widely used as sacrificial templates because of its porous structure and water-soluble properties. Additionally, as shown in Figure 13b, Kwon et al. [108] injected Ecoflex into the sugar template to obtain a highly compressible dielectric layer. The capacitive flexible pressure sensor with internal porous microstructure achieved an ultra-low pressure detection limit of 0.1 Pa, and its sensitivity were 0.601 kPa^−1^ (<5 kPa) and 0.077 kPa^−1^ (30–130 kPa).

Li et al. [109] prepared a MXene/chitosan (CS)/polyurethane (PU) sponge by dip coating. It was realized by alternately dipping and coating CS and MXene sheets on the skeleton of PU sponge, as shown in Figure 13c. Owning to the strong electrostatic interaction between MXene sheets and CS, MXene sheets were wrapped on the PU skeleton in a stable way, forming a 3-D conductive network on the PU skeleton. Due to the highly compressive resilience of the PU sponge and its polar interaction with the MXene sheets, the MXene/CS/PU sensor had high compressibility and stable piezoresistive response for stress of 245.7 kPa. In addition, the sensor had fast response time (19 ms), low detection limit (9 Pa), and good cycle stability (stable in 5000 compression release cycles). 

However, the sensitivity of pressure sensors with porous microstructures are generally not very high. In order to prepare flexible pressure sensors with both low density and high sensitivity, as shown in Figure 13d, Yang et al. [110] decided to combine the porous microstructure with pyramid microstructure to make full of the high sensitivity of it. So, they filled PS microspheres in the cavity of photolithographic Si template with concave pyramid shape, coated a layer of PDMS solution, and peeled off it after a certain pressure and heating treatment. Then PDMS dielectric layer with porous-pyramid structure could be obtained by dissolving PS microspheres with toluene. The capacitive pressure sensor corresponding to this microstructure had a sensitivity of 44.5 kPa^−1^ below 100 Pa.

Table 7 lists the performance summary of some sensors with porous microstructures. It is not difficult to find that the detection limits of these sensors are very low, which is very suitable for low pressure detection occasions. Coupled with its low density and high compressibility, the flexible pressure sensors with internal porous microstructures are attracting more and more researchers’ attention.

In conclusion, sensors with small shape factor microstructures (sharp bulge microstructure and micro fluctuation structure) generally have high sensitivity under low pressure, while sensors with large shape factor microstructures (wave/ridge microstructure) generally have a wide sensing range, but the sensitivity at low pressure is not as good as the former. In order to make up for the above defects of single and non-hierarchical microstructure, scientists have developed hierarchical microstructure and composite microstructure, so as to improve the sensitivity and sensing range of the sensor at the same time, so that the sensor has excellent linearity. What is more, researchers have also developed porous microstructure with low density and high compressibility according to people’s requirements for sensors with light weight, low detection limit, and other properties.

Therefore, different microstructure designs give different performances to the sensor. According to the requirements of device performances and application scenarios, researchers can make trade-offs and comparisons between sensor performance such as sensitivity, sensing range and detection limit, and choose to design different shapes of microstructures for the sensor.

## 4. Applications

Because the microstructure designs of materials can bring more excellent sensitivity, sensing range and flexibility to the flexible pressure sensors, they are widely used in various fields of society. First, they are designed as wearable devices to detect various force signals of the human body, such as pulse signals, bending or motion signals, sound signals, etc., so as to achieve the purpose of sports and health monitoring. Moreover, because of its high sensitivity and low detection limit, they can be applied to electronic skins to imitate human sensory function.

### 4.1. Sports, Health Monitoring and Wearable Devices

Human motions are complex, including larger human motions (such as finger, elbow, and knee bending) and smaller human motions (such as breathing, pulse, heart rate, expression, voice signal). Monitoring human movement can not only realize the transmission of human information, but also use them as health data for diagnosis and prevention of diseases. Because some micro-structured flexible pressure sensor has the characteristics of low detection limit or wide response range, it can detect the human body signal in each pressure range. What is more, most of the materials selected by the sensors have the advantages of good biocompatibility, so they are widely prepared into wearable devices for human movement and health monitoring.

**Figure 14:**
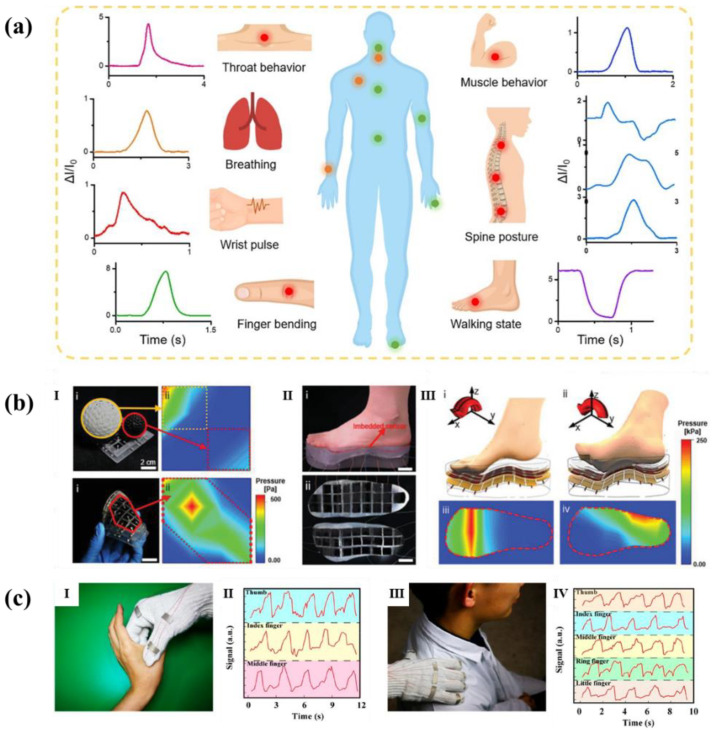
Microstructed flexible pressure sensors used in (**a**) monitoring the movement of the spine, throat and the body [60], (**b**) detecting foot pressure distribution [118], and (**c**) smart gloves [119].

Yang et al. [60] proposed a pressure sensor composed of multilayer polyvinylidene fluoride (PVDF)/polyaniline (PANI) film obtained by electrospinning and in-situ polymerization and rosette-hill-like substrate. As shown in Figure 14a, it can monitor the movement of the spine and throat, and can recognize the movement of the body, which can correspond to the physiological characteristics of the body. These detected human physiological signals contribute to clinical applications, such as the detection of respiratory diseases and the diagnosis and prevention of cardiovascular diseases. At the same time, they also provided important information for the monitoring of human activities and human health care, including quantitative motion measurement and spinal posture monitoring. 

Inspired by human skin, Sun et al. [118] developed a substrate combining folds with micro columns, and the sensor maintained good linearity (maximum 270 kPa) in a wide sensing range. Therefore, the sensor can detect physiological signals from the human body that span the pressure range of several orders of magnitude. Owing to sensor’s wide sensing range, it can be used to detect foot pressure distribution which is above a few hundred kilopascals. Scientists integrated a flexible sensor with 25 pixels into a 3-D printed insole. As shown in the mapped intensity profile (Figure 14b), the flexible sensor could accurately detect the foot pressure distribution depending on the foot posture. Ding et al. [119] developed a fabric-based pressure sensor assembled with cross-arranged pre-stretchable conductive yarn (PCY) weft and warp. The researchers directly weave the yarn into the gloves to form a pressure sensor. When a hand wore the smart glove under tension or pressure, different sensing signals were obtained. The smart gloves were used to massage the acupuncture points at the hand and shoulder. As presented in Figure 14c, the current signals of each finger were different due to tiny distinctions of massage intensity from each finger. According to the real-time signal change, the massage pressure can be monitored to avoid a burden for the skin and acupunctured point caused by over pressing. In addition, since the yarn can be woven into different sizes and patterns, the fabric assembled with the PCY sensor was suitable for a large area and real-time monitoring of coupling forces of pressure and tension in fine human motion and smart robots.

### 4.2. Tactile Perception and Electronic Skin

Flexible pressure sensor has made great progress in lots of fields of artificial intelligence, especially in the field of intelligent robots, because the electronic skin made of flexible pressure sensor is an indispensable part of it. However, with the development of electronic skin, the demands for the more excellent performances of flexible pressure sensor are higher and higher, and the structured flexible pressure sensor has better sensitivity, sensing range, and other performances, so it has been widely concerned by researchers.

**Figure 15:**
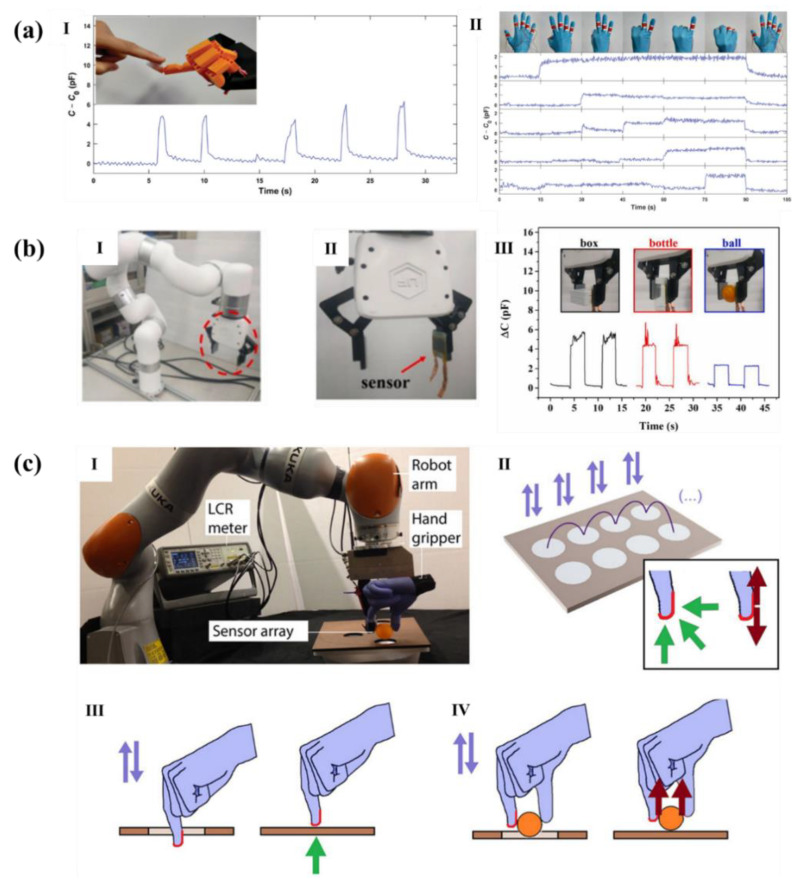
Micro structed flexible pressure sensors used in the electronic skin of robot.(**a**): (**I**) The sensor mounted on a robotic hand; (**II**) Five sensors mounted on a nitrile glove, which can be used to distinguish between different hand gestures. [120]. (**b**): (**I**) Digital photo of the robotic arm; (**II**) Magnified view of the claw with an attached sensor; (**III**) The capacitive responses when the claw holds the box, bottle, and ball [52]. (**c**): (**I**) E-skin sensor array mounted on an artificial hand and fixed on a gripper attached to the robot arm; (**II**) Typical test plate with holes; (**III**) The artificial hand is used to recognize the test plate with hole or not; (**IV**) A ping-pong ball was positioned between the two artificial fingers. Purple arrows show the movement executed downward, unless shear (tangential) force feedback (dark red arrow) was detected and prevented the entire execution of downward movement. [99].

Elsayes et al. [120] made a capacitive flexible sensor as electronic skin by using silver-nanowire-coated leaf skeletons as breathable and flexible electrodes and freeze-dried rose petals as the dielectric layer. They installed electronic skin on robot hands and gloves to monitor human gestures. As shown in Figure 15a, the electronic skin can detect when human fingers touch the manipulator repeatedly and recognize the gesture of each finger very accurately, indicating that the sensor can be used in practical application. Lin et al. [52] developed a composite dielectric layer with both porous fiber structure and micro cylindrical structure and prepared a capacitive flexible sensor. Researchers applied it to the robot arm to hold the box, glass bottle, or table tennis with the same 50 N clamping force, as shown in Figure 15b. Due to the change of contact area, the capacitance response was different, that is, the electronic skin can recognize different objects. Therefore, it had great potential to be applied to inanimate objects similar to manipulator. Boutry et al. [99] proposed a capacitive pressure sensor based on lithography template, which is composed of micro dome bottom electrode, intermediate dielectric layer, and pyramid top electrode. The scientists installed the sensor on an artificial hand fixed to the robot arm and used it as an electronic skin. As shown in Figure 15c, with the help of a multiplex acquisition platform, the artificial hand can place a light object on the table or make the object pass through a small hole by measuring the pressure and shear force on the sensor. Moreover, due to the high sensitivity of electronic skin, it showed excellent tactile sensing ability, which can allow the robotic device to interact with deformable and delicate objects, such as a fresh raspberry.

### 4.3. Other Applications

In addition to being studied in wearable devices and electronic skin, numerous flexible pressure sensors with excellent performances have also been reported to have application potential in smart city, human-computer interaction, education, and so on.

**Figure 16:**
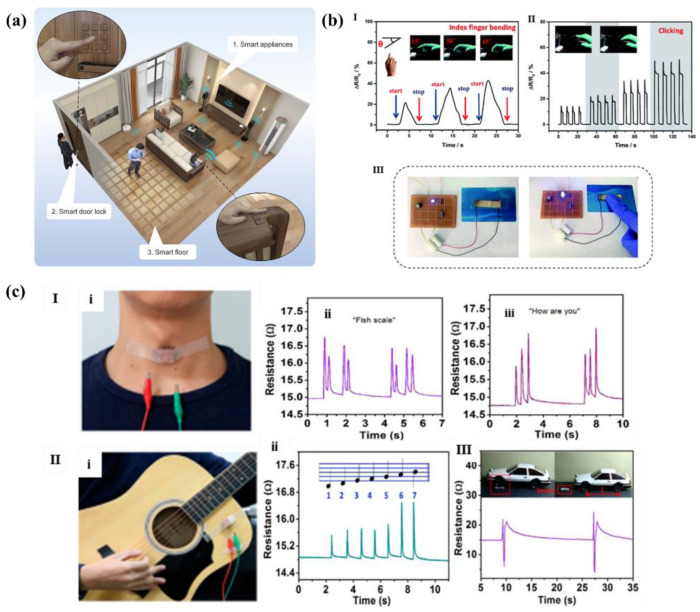
Micro structed flexible pressure sensors used in (**a**) smart home [121], (**b**) human-computer interaction [122], (**c**) speech recognition (**I**,**II**), and speed detection (**III**) [112].

Shi et al. [121] directly used natural materials as the dielectric layer of sensors to make full use of their microstructures. Wood is a common material in furniture and the treated wood has excellent performance, so the flexible wood-based triboelectric sensor can be highly integrated with various household facilities made of wood materials to build a smart home system. As shown in Figure 16a, with a simple signal processing circuit, a wood-based triboelectric self-powered sensor (WTSS)-based self-powered smart home control system that can remotely control household appliances and software was developed. This research demonstrated the promising applications of the environmentally friendly WTSS in constructing smart homes and smart cities, which will bring a great opportunity in the development of sustainable society in the future. 

Inspired by scorpion, Zhang et al. [122] developed an interlocking PDMS films with the negative and positive patterns of the microcrack arrays and made a flexible piezoresistive sensor with a sensitivity of 27.79 kPa^−1^ (0–2.4 kPa). As shown in Figure 16b, Scientists applied it to monitor the tapping of personal computer mouse, and the sensor showed a fast and reliable response to the tapping of fingers. Additionally, when the sensor was used as a flexible switch to control the brightness of the light-emitting diode (LED), it also exhibited a fast and repeatable response, with the brightness of the LED changing significantly. Therefore, researchers thought that flexible pressure sensors would have great development potential in the fields of human-computer interaction equipment, flexible touch screen, and so on.

Wu et al. [112] prepared a gold decorated 3-D microporous structure of PU sponge by ion sprinting, on which channel cracks were also skillfully designed. The sensor based on such Au/PU skeletons was equipped with excellent elasticity, fast response time (9 ms), and ultralow detection limit (0.568 Pa). As shown in Figure 16c, due to the low detection limit, the sensor can not only detect different words of pronunciation when attached to throw, but also detect the guitar notes by wave of string when scientists played fundamental formula of every note (“1 2 3 4 5 6 7”). Therefore, the sensor can be applied to speech recognition system for pronunciation calibration, recording, and noise detection. What is more, the sensor can also be used for large-scale motion detection, and scientists have applied it to the monitoring of the front and rear wheels of cars. Since the sensor is able to withstand the heavy pressure of the wheels, it can be used to calculate the vehicle speed, which shows a huge potential application in speed detection.

## 5. Conclusions

This paper summarizes the performance optimization strategies of flexible pressure sensors from the perspective of material microstructure design, analyzes the characteristics, advantages and disadvantages of different microstructures, and introduces the applications of micro-structured flexible pressure sensors in wearable devices, electronic skin, and other fields. It is obvious that the proper microstructure design of the sensor can effectively improve the sensitivity, sensing range, response time, and other characteristics of the sensor, or make the sensor lighter and more flexible to meet different use needs. The micro-structured sensor can become an important part of the next generation of wearable devices and electronic skin.

However, opportunities are always accompanied by challenges. It is apparent that sensitivity and sensing range are often a pair of contradictions. How to make the sensor have both these two characteristics is the primary problem that researchers need to face today. From the perspective of material microstructure design, microstructures with small shape factor, such as sharp bulges and micro fluctuations, can obtain better sensitivity with stress concentration effect under low pressure, while structures with large shape factor can obtain a wider sensing range. Layering a single structure or combining the two may give full play to the advantages of the sensor in these two aspects. In addition, with the development of science and technology, sensors should develop in the direction of higher sensitivity, wider sensing range, and other better performances. Researchers should not only continue to work hard to develop low-cost and smaller scale microstructure design technology, but also meet the needs of scientific and technological strength and economic benefits of social development, promoting the vigorous development of the flexible electronic industry.

## Figures and Tables

**Table 1:** The comparison of different types of flexible pressure sensors.

Type	Schematic Diagram	Sensing Principle	Characteristics
Piezoresistive	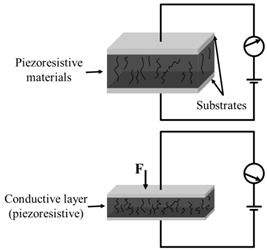	When under pressure, the resistance of the sensor will change based on the piezoresistive mechanism	High sensitivity Wide sensing range Simple structure and manufacturing technology Low cost Poor stability Lag effect
Capacitive	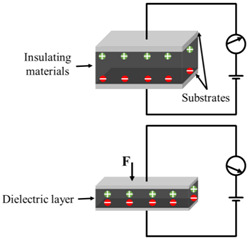	When under pressure, the dielectric constant or physical size of the dielectric layer changes, so that the capacitance of the sensor changes	High sensitivity Stable to temperature Mature manufacturing technology Low power consumption Highly susceptible to parasitic effects and electromagnetic interference
Piezoelectric	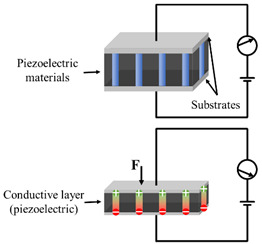	When under pressure, the piezoelectric material generates charge based on the inverse piezoelectric effect. This is due to the inherent dipole moment of piezoelectric materials. Pressure will deform the oriented non centrosymmetric crystal structure, resulting in the separation of electric dipole moment and voltage	High sensitivity Good dynamic response Self-power supply capacity Not suitable for static sensing Drift of sensor output over time
Triboelectric	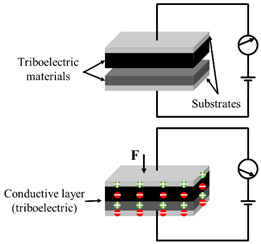	When under pressure, two materials with different friction polarities contact each other, and opposite charges are induced on both sides of the surface based on the triboelectric effect	High sensitivity Wide detection range Self-power supply capacity Simple manufacturing process Low cost Not suitable for static sensing

**Table 2:** The summary of sensor performances with sharp bulge microstructures.

Structure	Materials	Type	Sensitivity/Working Range	Detection Limit	Response Time	Reference
Pyramid	PDMS/rGO	Piezoresistive	5.53 kPa^−1^ (<0.1 kPa) 0.1 kPa^−1^ (0.1–1.4 kPa)	2 Pa	0.2 ms	[45]
	PDMS/poly(3,4–ethylenedioxythiophene–poly(styrenesulfonate) (PEDOT:PSS)/polyurethane dispersion(PUD)		4.88 kPa^−1^ (0.37–5.9 kPa)	37 Pa	0.2 ms	[39]
	PDMS/multiwalled carbon nanotubes (MWCNT)/Au/Ti/PET		9.95 kPa^−1^ (<0.1 kPa)		<200 ms	[53]
	PDMS/SWCNT		2760 kPa^−1^ (<0.4 kPa) 8655.6 kPa^−1^ (0.4–0.9 kPa) 1874.5 kPa^−1^ (>0.9 kPa)	7.3 Pa	<4 ms	[46]
	PDMS/PPy/Au		1907.2 kPa^−1^ (<0.1 kPa) 461.5 kPa^−1^ (0.1~1 kPa) 230.1 kPa^−1^ (1~1.9 kPa)	0.075 Pa	0.05 ms	[54]
	PDMS/vertical gold nanowire (v-AuNWs)		23 kPa^−1^ (<0.6 kPa) 0.7 kPa^−1^ (>0.6 kPa)		10 ms	[55]
Micro cone	PDMS/poly(methylmethacrylate)(PMMA)	Piezoresistive	2.5 kPa^−1^ (0–0.16 kPa) 0.2 kPa^−1^ (0.16–0.5 kPa)	15 Pa	20 ms	[56]
Lotus leaf-like sharp bulge	PPy/Ag	Piezoresistive	0.58 kPa^−1^ (0.3–0.4 kPa)			[47]
	PDMS/1–ethyl−3-methylimidazolium bis-(trifluoromethylsulfonyl)imide ([EMIM][NTF_2_])/AgNWs	Capacitive	2.09 kPa^−1^ (<80 kPa)			[57]
	PDMS/polystyrene (PS)/Au		0.815 kPa^−1^ (0–50 N)	17.5 Pa	38 ms	[58]
	PDMS/AgNWs/PI		1.2 k Pa^−1^ (<2 kPa)	0.8 Pa	36 ms 58 ms	[25]
Rose-like sharp bulge	PDMS/Cu–AgNWs	Piezoresistive	1.35 kPa^−1^ (<2 kPa) 0.1 kPa^−1^ (2–5 kPa)	2 Pa	36 ms 30 ms	[59]
	PDMS/PPy		70 kPa^−1^ (<0.5 kPa) 19 kPa^−1^ (0.5–2 kPa)	0.88 Pa	30 ms	[49]
	PDMS/polyvinylidene fluoride (PVDF)/polyaniline (PANI) fibers/Au		53 kPa^−1^ (58.4–960 Pa) 1.03 kPa^−1^ (0.96–5 kPa)	58.4 Pa	38 ms	[60]
	PDMS/indium tin oxide (ITO)/PET	Capacitive	0.055 kPa^−1^ (0.5–10 kPa)		<200 ms	[61]
Calathea Zebrine-like sharp bulge	colorless polymide (CPI)/AgNWs	Capacitive	54.31 kPa^−1^ (<0.5 kPa) 30.11 kPa^−1^ (0.5–10 kPa) 8.42 kPa^−1^ (10–40 kPa) 1.03 kPa^−1^ (40–115 kPa)		29 ms	[62]
	PDMS/polytetrafluoroethylene (PTFE)/AgNWs	Triboelectric	127.22 mV kPa^−1^ (<50 kPa)			[48]
Cilia	PDMS/Graphene/polyvinyl chloride (PVC)	Piezoresistive	4 kPa^−1^ (0–0.1 kPa) 0.25 kPa^−1^ (0.1–1 k Pa)	0.9 Pa		[63]
	PDMS/PVDF fiber	Capacitive	0.6 kPa^−1^ (0–7 kPa) 0.51 kPa^−1^ (7–15 kPa) 0.03 kPa^−1^ (15–50 kPa)	0.065 Pa	25 ms	[52]

**Table 3:** The summary of sensor performances with micro fluctuation structures.

Structure	Materials	Type	Sensitivity/Working Range	Detection Limit	Response Time	Reference
Micro dome	PDMS/Au	Piezoresistive	15 kPa^−1^ (<100 Pa) 2 kPa^−1^ (100–400 Pa)	4 Pa	100 ms	[64]
	PDMS/PMMA		0.64 kPa^−1^ (<400 Pa)	79 Pa	28 ms	[68]
	PDMS/CNT		1.82 kPa^−1^ (<2 kPa)	1 Pa	36 ms	[69]
	PDMS/Au		196 kPa^−1^ (<10 kPa) 12.8 kPa^−1^ (10–100 kPa)	0.5 Pa	25 ms	[70]
	PDMS/Graphene/PI		50.45 kPa^−1^ (<0.05 kPa) 4.35 kPa^−1^ (0.05~0.4 kPa)	0.209 Pa	39 ms	[71]
	PDMS/Carbon powder		124 kPa^−1^ (0–200 Pa) 0.39 kPa^−1^ (0.2–5 kPa)	2 Pa		[72]
	PDMS/Au	Capacitive	30.2 kPa^−1^ (<130 Pa) 0.47 kPa^−1^ (130 Pa–10 kPa)	0.7 Pa	25 ms	[73]
Ginkgo biloba leaf-like micro fluctuation	PDMS/Ag	Piezoresistive	5.9 kPa^−1^ (0–15 kPa)		42 ms 53 ms	[74]
	PDMS/MXene/PVA		164.93 kPa^−1^ (0~10 kPa) 403.46 kPa^−1^ (10~18 kPa)	0.88 Pa	99.3 ms	[65]
	thermoplastic polyurethane (TPU)/carbon black (CB)	Capacitive	1.194 kPa^−1^ (<1 kPa)	6.53 Pa	80 ms	[75]
Mimosa-like micro fluctuation	PDMS/Au	Piezoresistive	50.17 kPa^−1^ (0–0.07 kPa) 1.38 kPa^−1^ (0.2–1.5 kPa)		20 ms	[76]
Epipremnum aureum leaf-like micro fluctuation	PDMS/CNT/Graphene	Piezoresistive	19.8 kPa^−1^ (<0.3 kPa) 0.27 kPa^−1^ (0.3–6 kPa)	0.6 Pa		[66]
	PDMS/MWCNT		83.9 kPa^−1^ (<0.14 kPa) 0.4 kPa^−1^ (0.14–10 kPa)	0.5 Pa	90 ms 130 ms	[77]
	PDMS/Graphene		110 kPa^−1^ (<0.2 kPa) 3 kPa^−1^ (0.2–15 kPa) 0.26 kPa^−1^ (15–75 kPa)	0.2 Pa	<30 ms	[78]
Oleander-like micro fluctuation	PDMS/Ag	Piezoresistive	8.5 kPa^−1^ (<0.8 kPa) 0.2 kPa^−1^ (0.8–8.8 kPa)	1 Pa	40 ms	[44]
Silk-like micro fluctuation	PDMS/SWNTs	Piezoresistive	1.80 kPa^−1^ (<0.3 kPa)	0.6 Pa	<10 ms	[67]

**Table 4:** The summary of sensor performances with wave/ridge microstructures.

Structure	Materials	Type	Sensitivity/Working Range	Detection Limit	Response Time	Reference
Ridge	PDMS-polyurea (PUa)-1,3,5-triformylbenzene (TFB) _0.1_	Piezoresistive	8.7 kPa^−1^ (<6.1 kPa) 0.9 kPa^−1^ (6.1–29.1 kPa)	50 Pa	40 ms	[82]
	PDMS/PVA/H_3_ PO_4_	Capacitive	37.78 kPa^−1^ (<4 kPa) 16.81 kPa^−1^ (4–100 kPa) 4.69 kPa^−1^ (100–350 kPa)	0.32 Pa	23 ms	[79]
	PDMS/Ionic gel		92 kPa^−1^ (<0.4 kPa) 45.04 kPa^−1^ (0.4–20 kPa) 8.5 kPa^−1^ (20–100 kPa) 1.25 kPa^−1^ (100–300 kPa)	1 Pa	45 ms	[80]
Wave	PET/rGO/tape	Piezoresistive	5.77 kPa^−1^ (<0.49 kPa) 0.25 kPa^−1^ (0.49–9.8 Pa)	3 Pa	97 ms 98 ms	[81]
	PDMS/AgNWs	Capacitive	2.04 kPa^−1^ (<2 kPa) 0.57 kPa^−1^ (2–9 kPa)	7 Pa	100 ms	[83]

**Table 5:** The summary of sensor performances with hierarchical microstructures.

Structure	Materials	Type	Sensitivity/Working Range	Detection Limit	Response Time	Reference
Hierarchical sharp bulge	PDMS/AgNWs/PET	Piezoresistive	4.48 kPa^−1^ (<22 kPa) 0.86 kPa^−1^ (27–65 kPa)	3 Pa	7 ms	[84]
Hierarchical micro dome	PDMS/acrylonitrile butadiene styrene (ABS)	Piezoresistive	15.4 kPa^−1^ (<200 kPa)	16 Pa	20 ms	[88]
	PDMS/CNT	Capacitive	0.065 kPa^−1^ (<1700 kPa)		100 ms	[89]
Fold	PDMS/rGO	Piezoresistive	2.5 kPa^−1^ (10 Pa^−1^ kPa) 12.0 kPa^−1^ (1–50 kPa) 1051 kPa^−1^ (50–200 kPa) 470 kPa^−1^ (200–400 kPa)	10 Pa	150 ms	[90]
	PDMS/MWCNT		6.67 kPa^−1^ (0–20 kPa) 1.91 kPa^−1^ (20–100 kPa) 0.74 kPa^−1^ (100–270 kPa)	2 Pa	24 ms 30 ms	[91]
	PDMS/PEDOT:PSS		851 kPa^−1^ (<20 kPa)	34 Pa	0.15 ms	[92]
	PI/Mxene/Mo		3.844 kPa^−1^ (0–29 kPa) 12.095 kPa^−1^ (29–40 kPa)		26 ms	[93]
	Carbonized Silk/MoS		11.6 kPa^−1^ (0–0.25 kPa) 4.6 kPa^−1^ (0.25–3 kPa) 0.6 kPa^−1^ (3–20 kPa)	<70 Pa		[94]
	PDMS/PPy		19.32 kPa^−1^ (<0.8 kPa) 0.51 kPa^−1^ (>0.8 kPa)	1 Pa	20 ms	[95]
	PVA/H_3_ PO_4_	Capacitive	3302.9 kPa^−1^ (<10 kPa) 671.7 kPa^−1^ (10–100 kPa) 229.9 kPa^−1^ (100–360 kPa)	0.08 Pa	9 ms	[87]
	P(VDF-HFP)/[EMIM] [TFSI]/AgNWs		131.5 kPa^−1^ (<1.5 kPa) 11.73 kPa^−1^ (5–27.7 kPa)	1.12 Pa	43 ms	[86]
	PDMS/CNTs		9.55 kPa^−1^	<5 Pa	52 ms	[96]

**Table 6:** The summary of sensor performances with composite microstructures.

Structure	Materials	Type	Sensitivity/Working Range	Detection Limit	Response Time	Reference
Micro dome/grid	PDMS/MWCNT	Piezoresistive	0.90 kPa^−1^ (<600 Pa); 11.06 kPa^−1^ (600 Pa–10 kPa) 4.5 kPa^−1^ (10–300 kPa)			[98]
Micro dome/cone	PDMS/MWCNT		3.5 kPa^−1^ (0–218 kPa)		31 ms 52 ms	[101]
Micro groove/ridge	PDMS/PEDOT:PSS/Cu/Ag		62.56 kPa^−1^ (<0.7 kPa) 8.32 kPa^−1^ (0.7~6 kPa)			[103]
Micro elliptic cylinder/porous	PDMS/MWCNTs		10.805 kPa^−1^ (1 Pa^−1^ kPa); 2.015 kPa^−1^ (1–10 kPa)	1 Pa		[104]
Micro groove/sharp bulge	PDMS/MXene/AgNWs	Capacitive	2.08 kPa^−1^ (<1 kPa) 0.16 kPa^−1^ (1~100 kPa) 0.01 kPa^−1^ (100~600 kPa)		36 ms	[102]
Micro sharp bulge/porous	CPI/AgNWs		1.54 kPa^−1^ (<1 kPa) 0.014 kPa^−1^ (1~40 kPa) 0.068 kPa^−1^ (40~115 kPa)	0.6 Pa		[105]
Micro dome/pyramid	PDMS/CNT/PU		0.19 ± 0.07 kPa^−1^ (<1 kPa) 0.10 ± 0.01 kPa^−1^ (1~10 kPa) 0.04 ± 0.001 kPa^−1^ (10–20 kPa)			[99]
Micro pyramid/fold	PDMS/SWNT		0.7 kPa^−1^ (<25 kPa)		100 ms	[106]
Micro groove/sharp bulge	PDMS/Au		0.6 kPa^−1^ (<1 kPa) Maximum detection pressure is 40 kPa	4.5 Pa	180 ms 120 ms	[100]

**Table 7:** The summary of sensor performances with porous microstructures.

Structure	Materials	Type	Sensitivity/Working Range	Detection Limit	Response Time	Reference
Porous	3 D graphene microchannels (GMC)-PDMS/Au nanoparticles (AuNPs)	Piezoresistive	5.37 kPa^−1^ (<1 kPa) 1.56 kPa^−1^ (1–10 kPa) 0.5 kPa^−1^ (10–50 kPa)	4.4 Pa	20 ms	[111]
	PU/Au Sponge		0.059 kPa^−1^ (0–4.7 kPa) 0.096 kPa^−1^ (4.7 kPa–10.2 kPa) 0.122 KPa^−1^ (10.2 kPa–14.2 kPa)	0.568 Pa	9 ms	[112]
	PU/CS/MXene Sponge		0.014 kPa^−1^ (<6.5 kPa) −0.015 kPa^−1^ (6.5–85.1 kPa) −0.001 kPa^−1^ (>85.1 kPa)	9 Pa	19 ms	[109]
	PDMS/CNT Sponge		290.45 kPa^−1^ (0–25 kPa) 67.02 kPa^−1^ (25–270 kPa)		95 ms	[113]
	TPU/rGO Foam		0.0152 kPa^−1^ (20–500 kPa) 0.0007 kPa^−1^ (500~1940 kPa)		166 ms	[114]
	PDMS/Cu/Ni Nanofiber	Capacitive	0.171 kPa^−1^ (<5 kPa)		162 ms	[115]
	PDMS/BNF		0.854 kPa^−1^ (<0.5 kPa) 0.29 kPa^−1^ (0.5–2.1 kPa)	<1 Pa	50 ms	[107]
	PDMS/Cu/Ni		0.023 kPa^−1^ (<20 kPa)		155 ms	[116]
	Ecoflex/CNT		0.601 kPa^−1^ (<5 kPa) 0.077 kPa^−1^ (30–130 kPa)	0.1 Pa		[108]
	PDMS/ITO/PET		44.5 kPa^−1^ (<100 Pa)	0.14 Pa	50 ms	[110]
	PDMS/Ag nanoparticle (AgNP) Sponge	Piezoelectric	50 mV kPa^−1^ (<4 kPa) 1.8 mV kPa^−1^ (4~110 kPa) 0.8 mV kPa^−1^ (110–200 kPa)	4.1 Pa		[117]

## Data Availability

Not applicable.
